# Supplementary material for: Canadian Melanoma Conference Recommendations on High-Risk Melanoma Surveillance: A Report from the 14th Annual Canadian Melanoma Conference; Banff, Alberta; 20–22 February 2020
Source: Curr Oncol. 2021 May 27;28(3):2040–51. doi: 10.3390/curroncol28030189 (PMC8161801; doi:10.3390/curroncol28030189)
Supplement: Supplementary file 1 [file curroncol-28-00189-s001.zip › curroncol-1195382-supplementary.pdf]

Supplemental Materials A: Second iterative survey distributed to participants at the 19th annual CMC and designated melanoma clinicians across Canada.

1. 90% of respondents from our first survey agreed on intense surveillance among high-risk melanoma patients. What stage(s) constitute(s) high risk?
    - o stage II A; associated with 5 yr OS 93-94%
    - o stage II B/C; associated with 5 yr OS 82-86%
    - o stage III; associated with 5 yr OS 57-82%
    - o stages II B/C + III + IV
    - o stages III + IV
    - o treated stage IV (clinically NED)
2. For high-risk melanoma patients, what is an appropriate frequency for intense surveillance?
    - o twice a year (every 6 months)
    - o three times a year (every 4 months)
    - o four times a year (every 3 months)
3. The majority of surveillance protocols include a period of more intense surveillance followed by a less intense period (lower frequency of imaging and visits). Given the lack of data, do you feel it is necessary to follow patients beyond an intense period of surveillance?
    - o Yes
    - o No
4. What duration of less intense surveillance do you feel is appropriate?
    - o 3 years
    - o 4 years
5. 71% of respondents from the first survey agreed that systemic imaging should be performed every 6 months.
    - o 55% of respondents from the first survey agreed upon PET/CT as the modality of choice for systemic imaging every 6 months. Do you agree?
        - o Yes
        - o No
    - o PET/CT is not readily available across Canada, particularly in remote areas. Do you believe patients should travel to the nearest center to undergo PET/CT?
        - o Yes
        - o No

6. There was no consensus obtained on CNS imaging from our first survey. 43% agreed on annual brain MRI for all high-risk patients. At what frequency should a brain MRI be performed?
    - o every 3 months
    - o every 6 months
    - o annual
    - o other
7. Since MSLT II, the de-escalation of completion lymphadenectomy demonstrated that approximately 25% of sentinel node positive patients under observation progressed and required delayed surgery. In your opinion,
    - o Is it necessary to perform US surveillance of nodal basins on pathologically node negative patients? This is with respect to an overall false negative rate of 12.5% from SLNB
        - o Yes
        - o No

8. How often should US of nodal basins be performed if there is a positive sentinel node?
    - every 3 months
    - every 4 months
    - every 6 months
    - other
9. If regular PET/CT is obtained does this reduce the frequency of ultrasound required?
    - Yes
    - No
10. Serum analysis with LDH,
    - Is serum analysis with LDH necessary among all high-risk patients for surveillance?
        - Yes
        - No
    - At what frequency?
        - Every 3 months
        - Every 6 months
        - Annual

Supplemental Materials B: Third iterative survey at the 20th annual CMC conducted via sli.do s.r.o.

Participants were asked to respond in regards to their agreement to the following statements:

1. **Who**: High risk melanoma patients (Stage II B and C, Stage III and NED Stage IV) patients require close follow-up that should be undertaken by a combination of specialists with expertise in melanoma management including Surgeons, Medical Oncologists, Dermatologists, and Radiologists.
   - o Yes
   - o No
2. **Where:** While patients prefer that follow up be within a designated cancer center or melanoma clinic, follow up can be provided within any type of facility provided there is appropriate expertise (this may be especially true if special procedures or imaging is required like ultrasound of a node basin).
   - o Yes
   - o No
3. **When:** Physical examination with attention to the primary and nodal basins should be undertaken every 6 months for the first 2 years following surgical treatment and should be completed annually for the subsequent 3 years to a total of 5 years of follow up.
   - o Yes
   - o No
4. **What Modality:** Nodal U/S by experienced users was is recommended for surveillance of nodal basins every 4 months (sentinel node positive patients)
   - o Yes
   - o No
5. **Systemic Follow Up:** Recognizing that it is preferable to detect occult metastasis in asymptomatic patients, it is recommended that patients undergo systemic imaging every 6 months for years 1 – 2 and subsequently annually for a total of 5 years.
   - o Yes
   - o No
6. **Type of Systemic Imaging:** PET/CT scan is the preferred imaging modality, however, acknowledging that it is not widely available in many communities, CT Scan of the head, chest, abdomen and pelvis is acceptable every 6 months
   - o Yes
   - o No
7. **CNS Imaging**: Recognizing the significance of occult brain metastasis, imaging asymptomatic patients with Brain MRI annually is recommended.
   - o Yes
   - o No
8. Do you endorse the final follow up iteration presented?
   - o Yes
   - o No
